# NURSE PRACTITIONERS’ PRESCRIPTIVE AUTHORITY AND THE RURAL URBAN DISPARITY IN MORTALITY RATES

**DOI:** 10.1093/geroni/igz038.2084

**Published:** 2019-11-08

**Authors:** Nasim B Ferdows, Amit Kumar

**Affiliations:** 1 University of Southern California, Los Angeles, California, United States; 2 Northern Arizona University, Flagstaff, Arizona, United States

## Abstract

Although nurse practitioners (NPs) might be the main providers of primary care to some communities, different states pursue different regulations for NP’s practice authority. This study compared the trends in mortality rates and supply of physicians in states with different policies, using AHRF and CMF data. We categorized states based on their restrictive policies into: full, reduced, and restricted practice. We compared the trends in age-adjusted mortality rate and physician supply in rural and urban areas, and examined within-state changes in rural-urban difference in physician supply and mortality. Our results indicate that as the level of restrictive policy increased the rural-urban mortality gap increased while physician supply declined. Furthermore, regardless of increase or decrease in physicians supply disparity, rural-urban mortality disparity declined in full practice states, with a negative association between a decline in rural-urban physician supply disparity and decline in rural-urban mortality disparity in full or reduced practice states.

